# Supplementary material for: Stochasticity, determinism, and contingency shape genome evolution of endosymbiotic bacteria
Source: Nat Commun. 2024 May 29;15:4571. doi: 10.1038/s41467-024-48784-2 (PMC11137140; doi:10.1038/s41467-024-48784-2)
Supplement: Supplementary file 1 — Supplementary Information [file 41467_2024_48784_MOESM1_ESM.pdf]

Supplementary Material for Stochasticity, determinism, and contingency shape genome evolution of endosymbiotic bacteria

Bret M. Boyd[1*], Ian James[2], Kevin P. Johnson[3], Robert B. Weiss[4], Sarah E. Bush[2], Dale H. Clayton[2], Colin Dale[2].

[1] Center for Biological Data Science, Virginia Commonwealth University, Richmond, Virginia.[2] School of Biological Sciences, University of Utah, Salt Lake City, Utah. [3] Illinois Natural History Survey, Prairie Research Institute, University of Illinois, Champaign, Illinois. [4] Department of Human Genetics, University of Utah, Salt Lake City, Utah. [*] boydbm@vcu.edu

These authors contributed equally: Bret M. Boyd, Ian James.

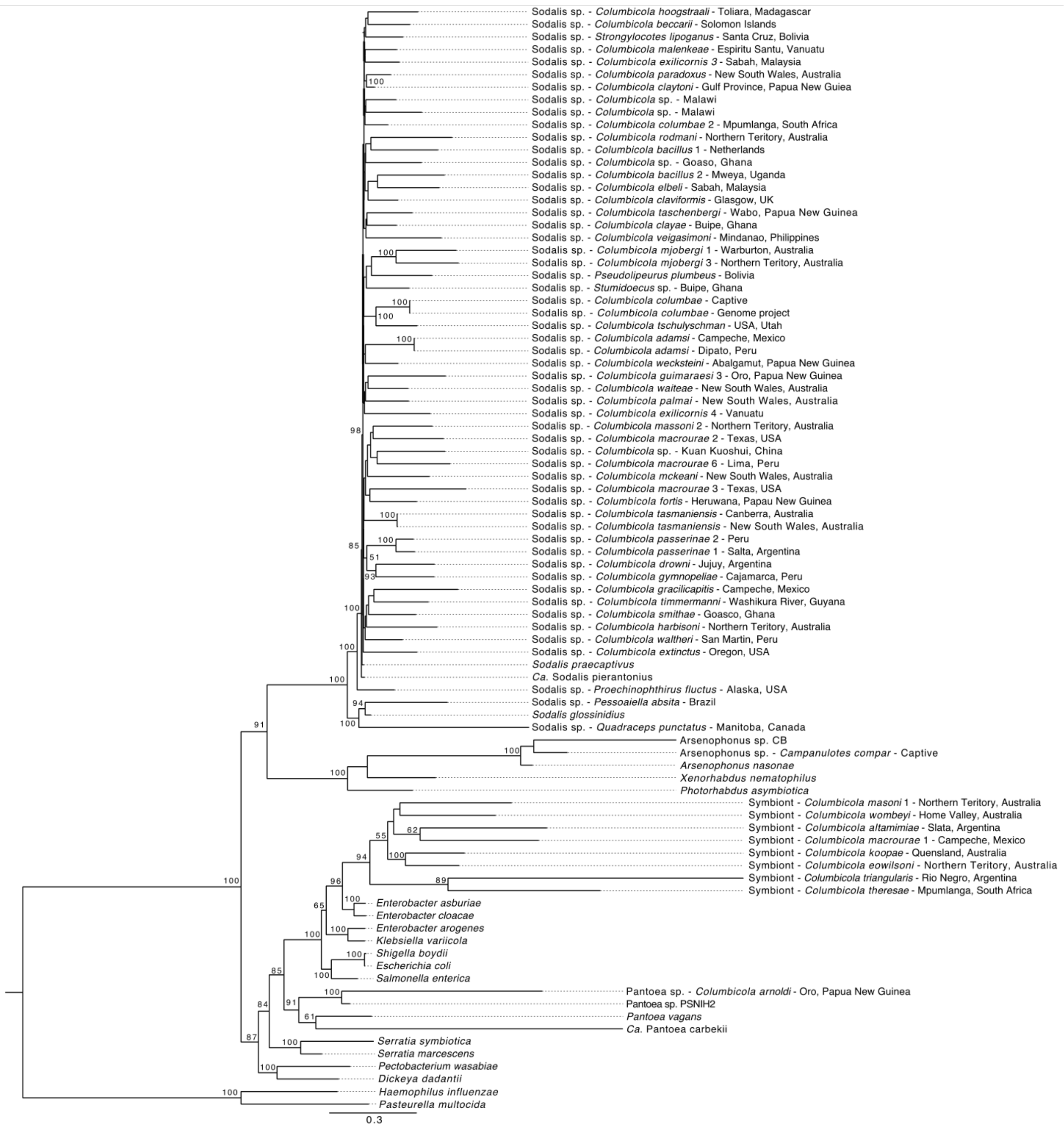

Fig. S1. Maximum-likelihood phylogeny of louse endosymbionts and representative Enterobacterales based on 13 single copy orthologs. Tree also presented in fig. 1a, but shown here with host and collection location described at tree tips. Tree with all bootstrap values and underlying DNA sequence alignments can be found in the data repository. Source data are provided as a Source Data file.

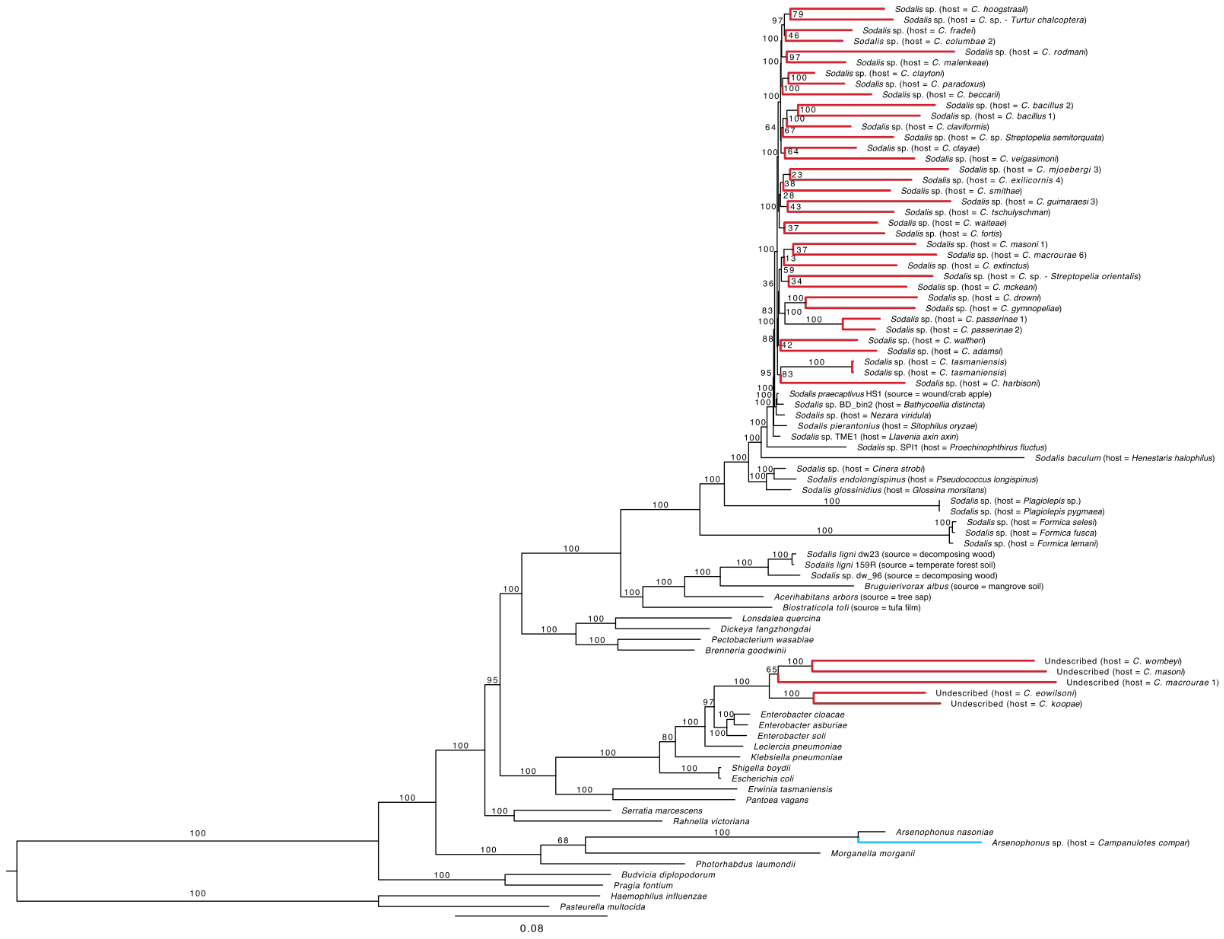

Fig. S2. Maximum-likelihood phylogeny of louse endosymbionts and representative Enterobacterales based on 241 single copy orthologs using the GTR-G model of base substitutions. Tree also presented in fig. 1a, but shown here with host and collection location described at tree tips. Tree with all bootstrap values and underlying DNA sequence alignments can be found in the data repository. Source data are provided as a Source Data file.

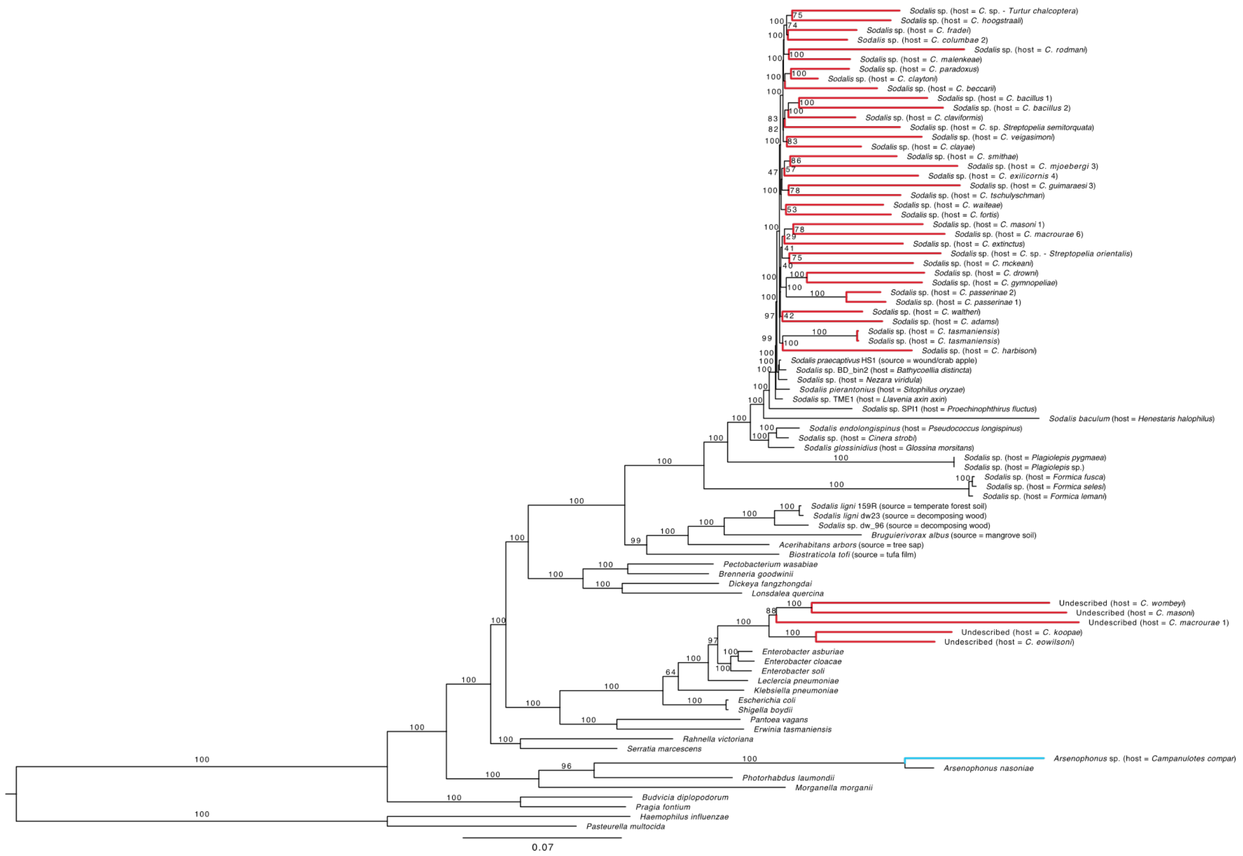

Fig. S3. Maximum-likelihood phylogeny of louse endosymbionts and representative Enterobacterales based on 241 single copy orthologs using selected time-reversible base substitution models. Tree also presented in fig. 1a, but shown here with host and collection location described at tree tips. Tree with all bootstrap values and underlying DNA sequence alignments can be found in the data repository. Source data are provided as a Source Data file.

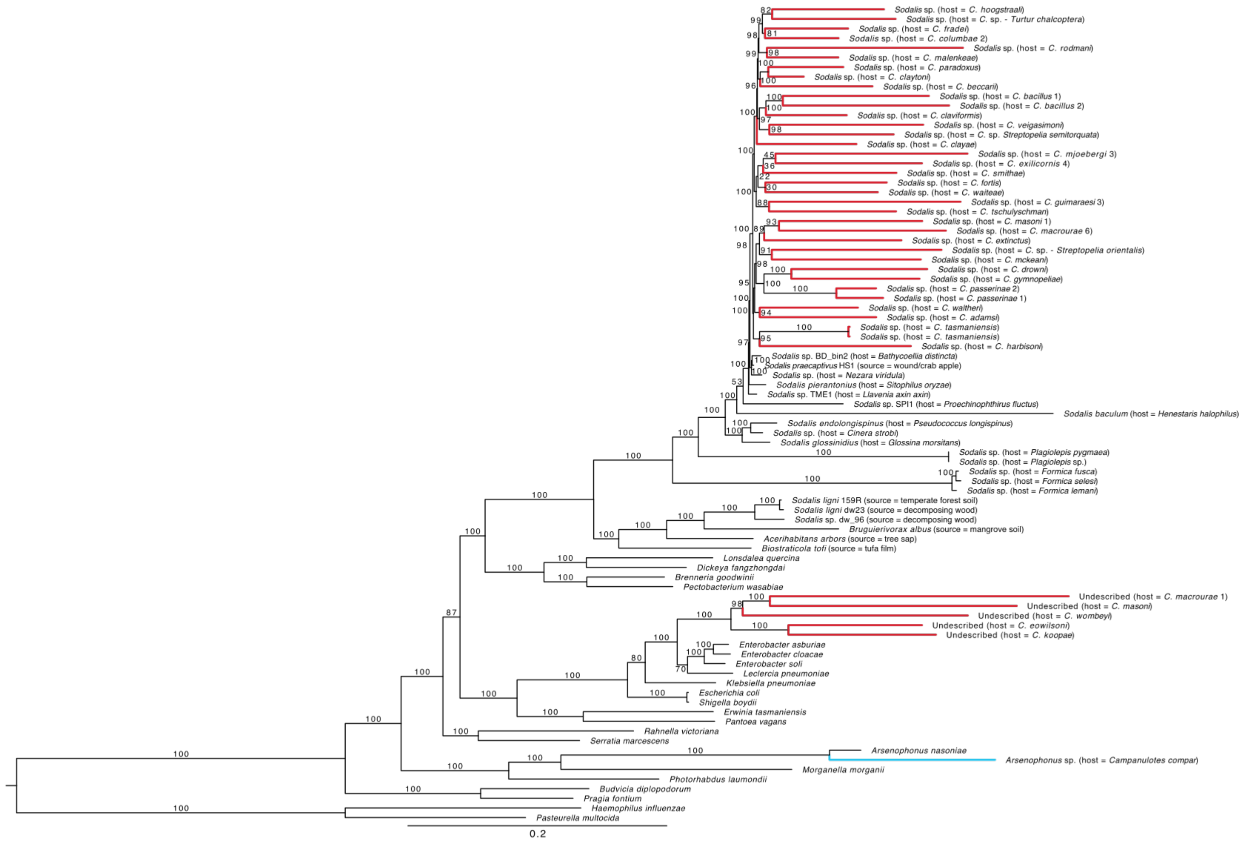

Fig. S4. Maximum-likelihood phylogeny of louse endosymbionts and representative Enterobacterales based on 241 single copy orthologs using selected amino acid base substitution models. Tree also presented in fig. 1a, but shown here with host and collection location described at tree tips. Tree with all bootstrap values and underlying amino acid sequence alignments can be found in the data repository. Source data are provided as a Source Data file.

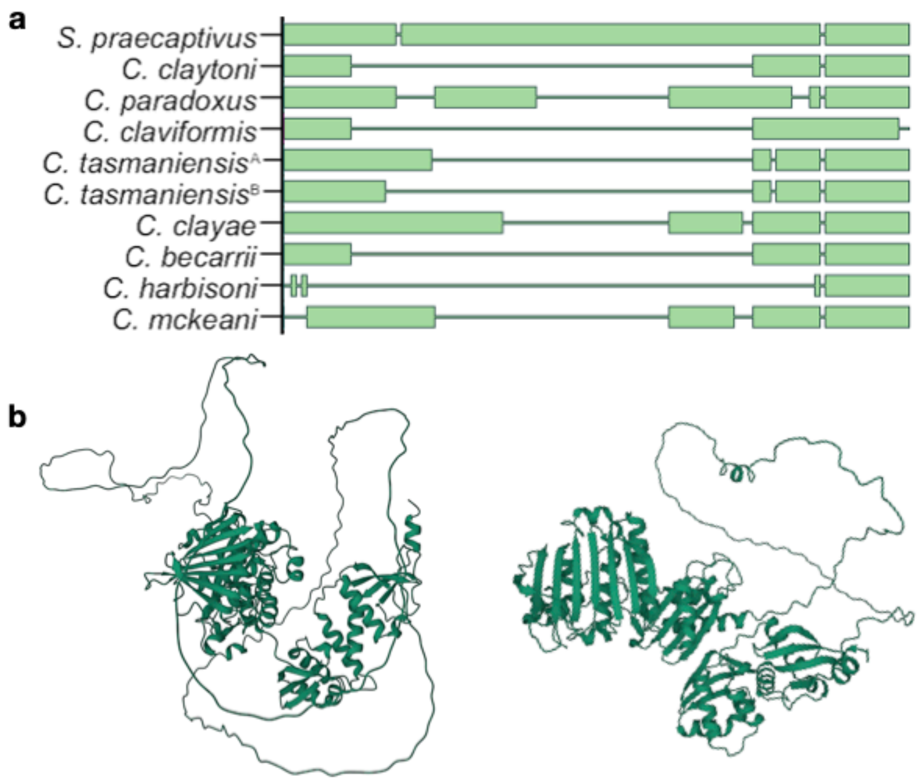

Fig. S5: MutL linker sequence and annotation. a, Alignment of the MutL disordered linker sequences found in *S. praecaptivus* (top) and those louse endosymbionts predicted to maintain intact copies of MutH. Green bars depict regions of sequence coverage. **b**, AlphaFold prediction of the structure of MutL in *S. praecaptivus* (left) and the *C. claytoni* symbiont (right). In each, the two domains of the MutL monomer are connected by a largely unstructured linker that is substantially larger in the *S. praecaptivus* homolog. Abbreviations: *C.* = *Columbicola*.

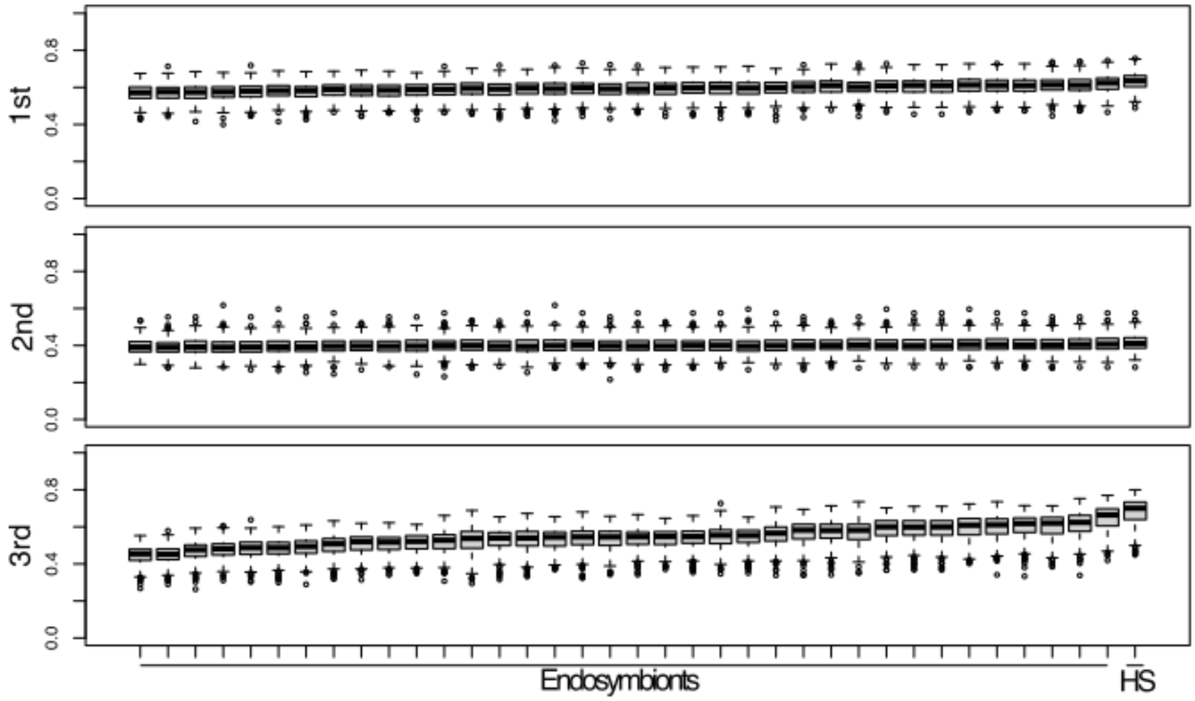

Fig. S6. Fraction of G+C bases by codon position and taxon in 297 universally conserved single copy orthologs in louse endosymbiont and *S. praecaptivus* HS1. Centerline, median; box limits, interquartile range; whiskers, outerquartile range; points, outliers. Source data are provided as a Source Data file.

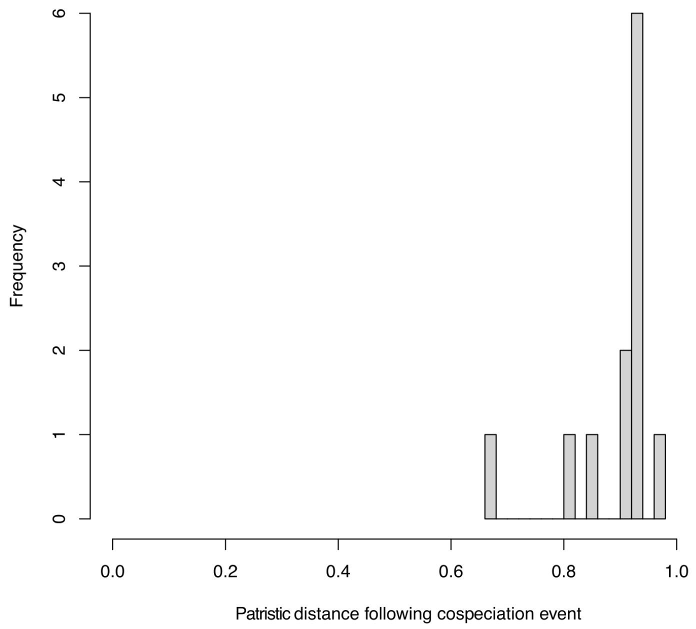

Fig. S7. Fraction of patristic distance consisting of branch following basal cospeciation event in louse endosymbionts. Distances obtained from phylogenetic tree present in Fig. 1b.

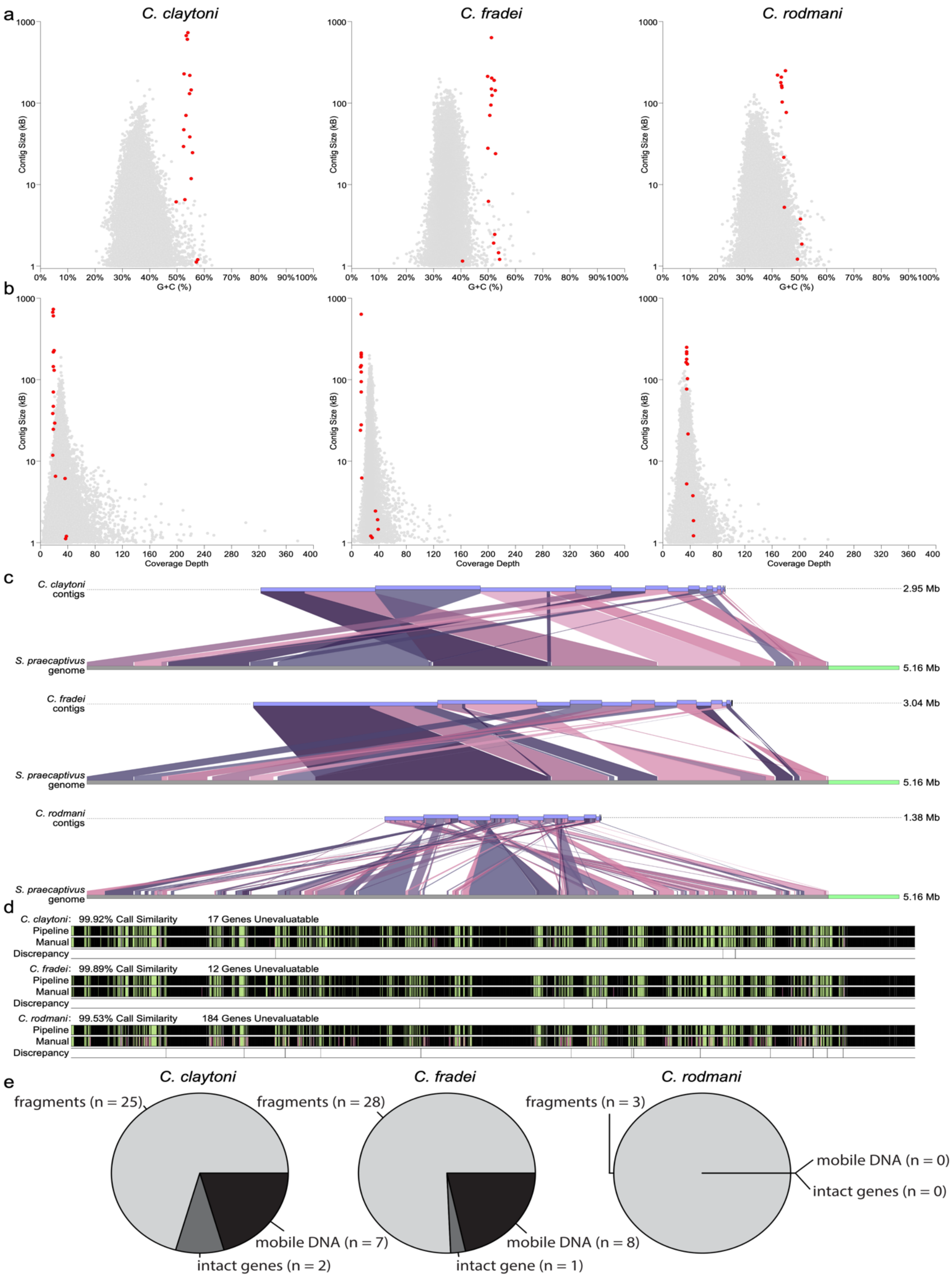
a
C. claytoni
C. fradei
C. rodmani
Contig Size (kB)
G+C (%)
b
Coverage Depth
c
C. claytoni contigs
2.95 Mb
S. praecaptivus genome
5.16 Mb
C. fradei contigs
3.04 Mb
C. rodmani contigs
1.38 Mb
d
C. claytoni: 99.92% Call Similarity 17 Genes Unevaluatable
Pipeline
Manual
Discrepancy
C. fradei: 99.89% Call Similarity 12 Genes Unevaluatable
C. rodmani: 99.53% Call Similarity 184 Genes Unevaluatable
e
fragments (n = 25)
mobile DNA (n = 7)
intact genes (n = 2)
fragments (n = 28)
mobile DNA (n = 8)
intact gene (n = 1)
fragments (n = 3)
mobile DNA (n = 0)
intact genes (n = 0)

Fig S8: Relationships between different methods for genome assembly. a, Relationships between G+C base content and contig size, comparing metagenomic assemblies of *C. claytoni, C. fradei and C. rodmani*. Predicted endosymbiont contigs have relatively similar G+C base content (for each endosymbiont) and are highlighted in red, while other contigs (comprising host sequences) are shown in grey. **b**, Relationship between sequencing depth and length of contigs from endosymbiont contigs shown in red in a. **c**, alignments of endosymbiont metagenomic contigs (top) and the genome of *S. praecaptivus* (bottom, plasmid shown in green). **d**, Matrices, analogous to those in Fig. 3a, depicting the near-identical gene inventories derived from the novel alignment-based approach, developed for this study (pipeline), versus the metagenomic assembly approach (manual). Genes that are determined by either approach to be intact are shown in green. Genes having ORFs spanning more than one contig or locally colinear block that cannot be evaluated (using the assembly-based approach) are shown in pink. Discrepancies in the annotation of intact genes between the two approaches are shown in black on a separate track labeled "Discrepancy" **e**, Representation of sequences derived from metagenomic assembly that were not aligned to *S. praecaptivus* by the Mauve algorithm, categorized as "fragments" (remnants of genes that are small in size), "mobile DNA" (corresponding to IS-element and phage sequences), and "intact genes". Abbreviations: *C.* = *Columbicola*. Source data are provided as a Source Data file.

Table S1: Endosymbiont identification, sample collection location, and host associations.

Avain host	Louse	Endosymbiont classification	Site of collection
Aplopelia larvata	*Columbicola fradei*	Sodalis	Malawi
Callorhinus ursinus	*Proechinophthirus fluctus*	Sodalis	USA: Alaska, St. Paul Island
Chalcophaps indica	*Columbicola guimaraesi 1*	undetermined	Vanuatu: Efate, Mangaliliu
Chalcophaps stephani	*Columbicola guimaraesi 3*	Sodalis	Papua New Guinea: Oro Province
Columba guinea	*Columbicola columbae 2*	Sodalis	South Africa: Mpumlanga
Columba leucomela	*Columbicola waiteae*	Sodalis	Australia: SW of Coffs Harbor, New South Wales
Columba livia	*Campanulotes compar*	Arsenophonus	Captive
Columba livia	*Columbicola columbae*	Sodalis	USA: IL, Champaign Co., Champaign
Columba livia	*Columbicola tschulyschman*	Sodalis	USA: Utah, Salt Lake City
Columba palumbus	*Columbicola claviformis*	Sodalis	United Kingdom: Glasgow
Columbina cruziana	*Columbicola passerinae 2*	Sodalis	Peru
Columbina picui	*Columbicola passerinae 1*	Sodalis	Argentina: Salta
Crypturellus tataupa	*Pseudolipeurus plumbeus*	Sodalis	Boliva
Ducula bicolor	*Columbicola wolffhuegeli*	undetermined	Australia: Northern Teritory
Ducula pacifica	*Columbicola malenkeae*	Sodalis	Vanuatu: Espiritu Santu
Ducula rufigaster	*Columbicola claytoni*	Sodalis	Papua New Guinea: Gulf Province
Gallicolumba beccarii	*Columbicola beccarii*	Sodalis	Solomon Islands: Makira Island, 17.5km S Kira Kira
Geopelia cuneata	*Columbicola mjoebergi 1*	Sodalis	Australia: W. of Warburton
Geopelia humeralis	*Columbicola rodmani*	Sodalis	Australia: Northern Teritory
Geopelia placida	*Columbicola mjoebergi 3*	Sodalis	Australia: 45km East of Darwin, Nothern Teritory
Geophaps plumifera	*Columbicola wombeyi*	Enterobacter	Australia: 7.7 km N of Home Valley Station
Geophaps scripta	*Columbicola koopae*	Enterobacer	Australia: Queensland, Mittchell River
Geophaps smithii	*Columbicola eowilsoni*	Enterobacter	Australia: Pine Creek, Northern Teritory
Geotrygon frenata	*Columbicola waltheri*	Sodalis	Peru: San Martin
Geotrygon montana	*Columbicola macrourae 1*	Enterobacter	Mexico: Campeche
Lamprotomis purpureus	*Stumidoecus sp.*	Sodalis	Ghana: Buipe
Larus argentatus	*Quadraceps punctatus*	Sodalis	Canada, Manitoba
Leptotila jamaicensis	*Columbicola gracilicapitis*	Sodalis	Mexico: Campeche
Leptotila rufaxilla	*Columbicola timmermanni*	Sodalis	Guyana: Washikura River
Leucosarcia melanoleuca	*Columbicola palmai*	Sodalis	Australia: New South Wales, Cordeaux Rd, Dapto
Lopholaimus antarcticus	*Columbicola paradoxus*	Sodalis	Australia: SW of Coffs Harbor in New Sout Wales
Macropygia amboinensis	*Columbicola exilicornis 1*	undetermined	Papua New Guinea: Eastern Highlands Prov.
Macropygia mackinlayi	*Columbicola exilicornis 4*	Sodalis	Vanuatu
Macropygia nigrirostris	*Columbicola arnoldi*	Pantoea	Papua New Guinea: Oro Province
Macropygia ruficeps	*Columbicola exilicornis 3*	Sodalis	Malaysia: Sabah
Metriopelia aymara	*Columbicola altamimiae*	Enterobacter	Argentina: Salta
Metriopelia ceciliae	*Columbicola gymnopeliae*	Sodalis	Peru: Cajamarca
Metriopelia melanoptera	*Columbicola drowni*	Sodalis	Argentina: Jujuy
Ocyphaps lophotes	*Columbicola mckeani*	undetermined	Australia: New South Wales, Mt. Hope
Opisthocomus hoazin	*Pessoaiella absita*	Sodalis	Brazil
Otidiphaps nobilis	*Columbicola fortis*	Sodalis	Papua New Guinea: Herowana
Patagioenas fasciata	*Columbicola extinctus*	Sodalis	USA: Oregon, Corvalis
Patagioenas oenops	*Columbicola adamsi*	Sodalis	Peru: Dpto. Cajamarca
Patagioenas picazuro	*Columbicola triangularis*	Enterobacter	Argentina: Rio Negro
Patagioenas speciosa	*Columbicola adamsi*	Sodalis	Mexico: Campeche
Petrophassa albipennis	*Columbicola masoni 1*	Enterobacter	Australia: Northern Teritory
Petrophassa rufipennis	*Columbicola masoni 2*	Sodalis	Australia: Kakadu National Park, Norhtern Teritory
Phapitreron leucotis	*Columbicola veigasimoni*	Sodalis	Philippines: Mindanao
Phaps chalcoptera	*Columbicola tasmaniensis*	Sodalis	Australia: NSW
Phaps elegans	*Columbicola tasmaniensis*	Sodalis	Australia: Canberra, Kingston/Manuka area
Phaps histrionica	*Columbicola harbisoni*	undetermined	Australia: Borclay Taselands, Northern Teritory
Ptilinopus rivoli	*Columbicola wecksteini*	Sodalis	Papua New Guinea: Abalgamut Camp
Reinwardtoena reinwardtsi	*Columbicola taschenbergi*	Sodalis	Papua New Guinea: Wabo
Rhynchotus rufescens	*Strongylocotes lipogonus*	Sodalis	Bolivia:Santa Cruz
Streptopelia capicola	*Columbicola theresae*	Enterobacter	South Africa: Mpumlanga
Streptopelia decaocto	*Columbicola bacillus 1*	Sodalis	Netherlands
Streptopelia decipiens	*Columbicola bacillus 2*	Sodalis	Uganda: Mweya
Streptopelia lugens	*Columbicola orientalis*	undetermined	Malawi
Streptopelia orientalis	*Columbicola* sp.	Sodalis	China: Kuan Kuoshui, Guizhou Provence
Streptopelia picturata	*Columbicola hoogstraali*	Sodlais	Madagascar: Toliara
Streptopelia semitorquata	*Columbicola sp.*	Sodlais	Ghana: Goaso
Treron vernans	*Columbicola elbeli*	Sodalis	Malaysia: Sabah
Treron waalia	*Columbicola clayae*	Sodalis	Ghana: Buipe
Turtur brehmeri	*Columbicola smithae*	Sodalis	Ghana: Goaso
Turtur chalcospilos	*Columbicola* sp.	Sodalis	Malawi
Turtur tympanistria	*Columbicola carrikeri*	undetermined	Malawi
Zenaida asiatica	*Columbicola macrourae 2*	Sodalis	USA: Texas, Las Palomas Wildlife Refuge
Zenaida macroura	*Columbicola macrourae 3*	Sodalis	USA: Texas, Las Palomas Wildlife Refuge
Zenaida meloda	*Columbicola macrourae 6*	Sodalis	Peru: Lima

**C. adamsi and C. tasmaniensis* were each sampled from two different dove species. Endosymbiont classification is based on preliminary pairwise comparisons with representative bacterial genomes.

SUPPLEMENTARY METHODS:

Phylogenomics:

Data sources: Predicted protein coding sets were downloaded from NCBI Genome Assembly database (https://www.ncbi.nlm.nih.gov/assembly). We favored CDS described in the RefSeq annotation, but accepted the GenBank annotation when the RefSeq annotation was not available. To sample critical taxa for which predicted CDS were not available on NCBI, we downloaded either un-annotated contigs from NCBI or the raw sequence reads from the NCBI short-read archive (https://www.ncbi.nlm.nih.gov/sra). In the case where we obtained an assembly lacking an annotation, we generated an annotation using RAST[1-2]. When raw reads were utilized for whole genome assembly, we prepared the reads using fastp v0.23.2 and preformed a *de novo* assembly using metaSPAdes (v.3.14.0)[3-4]. We then identified candidate contigs belonging to the endosymbiont genome using NCBI BLASTx v2.10.0+, comparing to a custom database composed of representative Enterobacteriaceae[5]. These putative endosymbiont contigs were then annotated using RAST or MiGa[6].

Ortholog identification: OrthoFinder v2.3.14 was used to identify orthologous gene groups among the newly assembled and downloaded gene sets[7]. From the resulting orthogroups, we identified each group that contained one of the 297 single-copy-orthologs used in our original *Sodalis* specific phylogenomic analysis. We then examined summary statistics, removing two newly downloaded species, due to poor representation in the focal 297 gene set. This left us with 48 newly added taxa, in addition to the 32 existing endosymbionts and *Sodalis praecaptivus* used in the targeted phylogenomic analysis of *Sodalis*. Next, we filtered the orthogroups, rejecting any individual species contribution to orthogroup that contained a paralog (i.e. one species contributed more than one locus). Finally, if four or more taxa were not represented in the filtered groups (i.e. they never contributed a gene or had their contribution removed due to paralogy), whole ortholog groups were rejected. This left us with 241 single-copy-orthologs, representing 95% or more of the taxa; effectively minimizing missing data, while providing many loci for phylogenetic estimation.

Alignment: Genes within orthogroups were then aligned as translated amino acid sequences using MUSCLE (v.3.8.31), and converted back to nucleotide[8]. Alignments were filtered using ClipKit (v.2.1.1)[9].

Comparative Genomics:

Calculation of Relationship Strength: The strength of direct or reciprocal/alternating relationships between strings was computed using the function $0.5E + \frac{0.5}{17}\sqrt{(H-17)^2}$, where $0.5E$ is the lesser of two Shannon Entropies (having a maximum possible value of 1) multiplied by 0.5 so that it contributes to one half of the relationship strength value. The other half of the relationship strength value is derived from the Hamming distance between strings, which corresponds to the strength of the direct or reciprocal/alternating match. However, because Hamming distances can be used to determine the strength of both direct and reciprocal/alternating matches $\sqrt{(H-17)^2}$ is used to transform values to a scale from 0 to 17, regardless of whether the relationship is direct or reciprocal/alternating.

Validation of Genome Assembly and Annotation Pipeline: The alignment and annotation pipeline was validated by generating *de novo* assemblies using metaSPAdes from sequence reads from the *C. claytoni*, *C. fradei* and *C. rodmani* endosymbionts using default parameters[10]. The *C.*

claytoni sample was chosen because it has the largest endosymbiont genome and is therefore anticipated to have a large quotient of pseudogenes that maintain the lowest level of sequence divergence from *S. praecaptivus* orthologs. Additionally, *C. fradei* was selected because its sequence reads have the lowest ratio of endosymbiont:host representation in the read library, resulting in a low depth of coverage in an assembly. Finally, *C. rodmani* was selected because its genes are anticipated to have the highest overall level of sequence divergence relative to *S. praecaptivus* orthologs, consistent with the fact that phylogenetic analyses reveal that it maintains the highest patristic distance from *S. praecaptivus*. The *C. claytoni*, *C. fradei* and *C. rodmani* assemblies yielded 55,314, 17,650 and 35,211 contigs, with median sizes of 2,084 bp, 6,351 bp and 2,647 bp respectively. BLASTN and BLASTX[11] searches were then performed, using the nr/nt and nr databases respectively, to identify all contigs sharing sequence identity with *S. praecaptivus*. In total, 17, 18 and 13 contigs were identified with size ranging from 1-728 kbp, 1-1,169 kbp and 1-247 kbp, comprising the genomes of the *C. claytoni, C. fradei* and *C. rodmani Sodalis*-allied endosymbionts (Fig. S8a-b). These contigs were concatenated and then aligned using the Mauve algorithm with default parameters to the genome of *S. praecaptivus* (Fig. S8c). Intact protein-coding genes were identified by manual inspection, (guided by the Mauve alignments) on the basis of similarity of the best predicted translation of the region aligning to each gene in *S. praecaptivus* including up to 25 bp of flanking sequence at the 3' and 5' ends of each candidate ORF (to account for alternative start and stop codons). Genes were designated as intact if their best fit translation shared at least 89.79%, 83.13%, and 64.75% similarity for *C. claytoni*, *C. fradei* and *C. rodmani* endosymbionts, respectively, consistent with cutoff values estimated previously using the expectation maximization approach described in Methods (see section "Genome Sequences Annotation" in the associated publication). Genes having ORFs that spanned more than one contig or locally colinear block (as defined in Mauve) were treated as missing data. In cases where the status of a gene could be identified in the metagenomic assembly-based approach, there was greater than 99% agreement between the inventories of intact genes derived from the alignment-based pipeline and the metagenomic assembly-based approach (Fig. S8d).

A small number of orphaned regions within the metagenomic contigs did not align to the *S. praecaptivus* genome using Mauve. We compared these regions to proteins available in the NCBI non-redundant (nr) protein database (database downloaded on March 9, 2022) using BLASTX, finding 47%, 31%, and 83%, in *C. claytoni*, *C. fradei* and *C. rodmani* endosymbiont respectively, of orphaned regions returned a significant hit ($e<0.1^{-5}$) to an *S. praecaptivus* protein. We filtered out sequences that returned a significant hit to *S. praecaptivus* genome, assuming that the regions share orthologs with *S. praecaptivus*, but failed to align. Of the remaining fragments, we identified open reading frames (ORFs) and isolated the longest ORFs (up to a total of 10 ORFs). We then compared these ORFs to proteins available in the nr database (as translated amino acids) using BLASTP. Again, some of the ORFs returned a significant hit to a *S. praecaptivus* protein and were excluded. We then evaluated the remaining ORFs based on the BLAST hit. The results indicate that the gene inventories of the *C. claytoni*, *C. fradei* and *C. rodmani* endosymbionts are largely subsets of *S. praecaptivus*, with the exception of small gene fragments (likely pseudogenes) and sequences derived from mobile genetic elements (IS-elements and phages), which are known to accumulate in the genomes of endosymbionts[12] (Fig. S8e). Aside from those, only two novel proteins were discovered (two copies of one in the endosymbiont of *C. claytoni* and one in the *C. fradei* endosymbiont), predicted to encode a metallochaperone and a PhoX-family phosphatase, respectively. Taken together, these validation efforts provide support for the notion that our custom (alignment-based) pipeline yields consistent and accurate annotation of the feather louse

endosymbiont genomes in our study, regardless of their patristic distance from *S. praecaptivus* and the depth of sequencing coverage of the endosymbiont genome. Furthermore, based on these validations the feather louse endosymbionts do not maintain significant genetic functionality beyond that encoded by *S. praecaptivus*.

Finally, to provide maximum accessibility to our data, we generated metagenomic assemblies using the sequence reads derived from all remaining lice used in our study, employing the same metaSPAdes approach. All contigs from each assembly sharing sequence identity with the *S. praecaptivus* whole genome sequence were identified and binned, yielding at least 1-Mb of sequence for each sample with the exception of *C. adamsi*, whose assembly yielded only a few symbiont contigs of small size due to low sequence coverage. Therefore, symbionts contigs from all samples except *C. adamsi*, were uploaded (https://www.ncbi.nlm.nih.gov/genome/).

Color Palettes and Maps: Figures in both the main paper and supplementary information use color palettes and maps derived from ColorBrewer 2.0[13], an online CIELAB gradient making tool (https://davidjohnstone.net/lch-lab-colour-gradient-picker), colorCET (colorcet.com)[14] and Scientific colour maps (version 8.01)[15] to minimize visual distortion and increase accessibility.

References

1. Aziz, R. K. et al. The RAST Server: Rapid annotations using subsystems technology. *BMC Genomics*. 9:75 (2008)
2. Overbeek, R., et al. The SEED and the rapid annotation of microbial genomes using subsystem technology (RAST). *Nucl. Acid. Res*. 42:D206-D214 (2013)
3. Chen, S., et al. fastp: an ultra-fast all-in-one FASTQ preprocessor. *Bioinf*. 34:i884-i890 (2018)
4. Bankevich, A., et al. SPAdes: A new genome assembly algorithm and its applications to single-cell sequencing. *J. Compu. Biol.* 19:455-477 (2012).
5. Altschul, S. F., Gish, W., Miller, W., Myers, E. W. & Lipman, D. J. Basic local alignment search tool. *J. Mol. Biol.* **215**, 403–410 (1990).
6. Rodriguez-R, L. M., et al. The microbial genome atlas (MiGA) webserver: taxonomic and gene diversity analysis of Archea and Bacteria at a whole genome level. *Nucl. Acid Res.* 46:W282-W288 (2018).
7. Emms, D. M., Kelly, S. OrthoFinder: phylogenetic orthology inference for comparative genomics. *Genome Biol*. 20:238 (2019).
8. Edgar, R. C. MUSCLE: multiple sequence alignment with high accuracy and high throughput. *Nucl. Acids Res.* **32**, 1792–1797 (2004).
9. Steenwyk, J. L., et al. ClipKIT: A multiple sequence alignment trimming software for accurate phylogenomic inference. *PLoS Biol.* 18:e3001007 (2020).
10. Nurk, S., Meleshko, D., Korobeynikov, A. & Pevzner, P. A. metaSPAdes: a new versatile metagenomic assembler. *Genome Res*. **27**, 824–834 (2017).
11. Altschul, S. F., Gish, W., Miller, W., Myers, E. W. & Lipman, D. J. Basic local alignment search tool. *J. Mol. Biol.* **215**, 403–410 (1990).
12. Siguier P, Gourbeyre E, Chandler M. Bacterial insertion sequences: their genomic impact and diversity. *FEMS Microbiology Reviews*. **5**, 865–891 (2014).
13. Brewer, C. and Harrower, M. ColorBrewer 2.0. (2012).
14. Kovesi, P. Good colour maps: how to design them. arXiv 1509.03700. (2015).
15. Crameri, F. Scientific colour maps (v.8.0.1). zenodo.org (2023).
