# Supplementary figures and images for: Stochasticity, determinism, and contingency shape genome evolution of endosymbiotic bacteria
Source: Nat Commun. 2024 May 29;15:4571. doi: 10.1038/s41467-024-48784-2 (PMC11137140; doi:10.1038/s41467-024-48784-2)

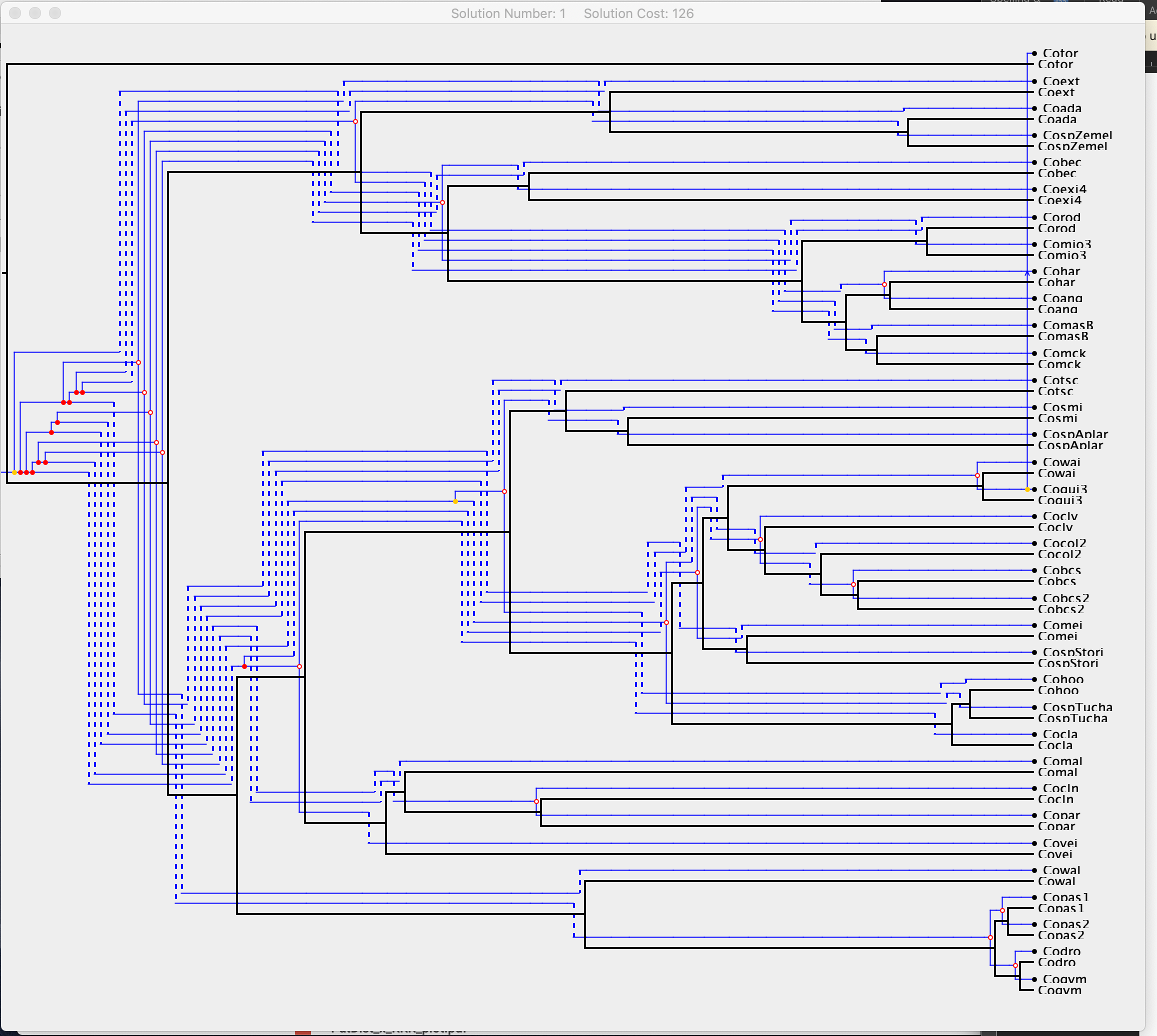

Supplement: Supplementary file 4 — Source Data [file 41467_2024_48784_MOESM4_ESM.zip › FigureSourceData/Figure1_SourceData/Fig1b_JANESolution.png]
